# Optimal threshold of three-dimensional echocardiographic fully automated software for quantification of left ventricular volumes and ejection fraction: Comparison with cardiac magnetic resonance disk-area summation method and feature tracking method

**DOI:** 10.1371/journal.pone.0211154

**Published:** 2019-01-28

**Authors:** Victor Chien-Chia Wu, Tetuji Kitano, Yosuke Nabeshima, Kyoko Otani, Pao-Hsien Chu, Masaaki Takeuchi

**Affiliations:** 1 Department of Laboratory and Transfusion Medicine, University of Occupational and Environmental Health Hospital, Kitakyushu, Japan; 2 Division of Cardiology, Chang Gung Memorial Hospital, Linkou Medical Center, Taoyuan City, Taiwan; 3 Second Department of Internal Medicine, University of Occupational and Environmental Health, School of Medicine, Kitakyushu, Japan; Faculty of Medical Science - State University of Campinas, BRAZIL

## Abstract

**Aims:**

Novel fully automated left chamber quantification software for three-dimensional echocardiography (3DE) has a potential for reliable measurement of left ventricular (LV) volumes and ejection fraction (LVEF). However, the optimal setting of global LV endocardial border threshold has not been settled.

**Methods and results:**

We performed LV volumes and LVEF analysis using fully automated left chamber quantification software (Dynamic HeartModel^A.I.^, Philips Medical Systems) in 65 patients who had undergone both 3DE and cardiac magnetic resonance (CMR) examinations on the same day. We recorded LV end-diastolic volume (LVEDV) and LV end-systolic volume (LVESV) according to the change in LV global border threshold settings from 0-point to 100-point with each increment of 10-point. These values were compared to the corresponding values of CMR with disk-area summation method and feature tracking (FT) method. Coverage probability (CP) was calculated as an index of accuracy and reliability. Fully automated software provided LV volumes and LVEF in 57 patients (Feasibility: 88%). LVEDV and LVESV increased steadily according to the increase in border threshold and reached minimal bias when border threshold setting was 80 against CMR disk-summation method and 90 against CMR FT method. Corresponding CP of LVEF was 0.74 and 0.84 against disk-area summation method and FT method.

**Conclusions:**

With CMR values as a reference, LV endocardial border threshold value can be set around 80 to 90 with the same number of LV end-diastole and end-systole threshold to approximate LVEDV, LVESV and LVEF with clinically acceptable CP values of LVEF.

## Introduction

Left ventricular (LV) ejection fraction (LVEF) using transthoracic two-dimensional echocardiography (2DE) is still one of the most important cardiac metrics to quantify LV function. Several cut-off values of LVEF has been used to select candidates for implantable cardiac defibrillator [1] and to determine discontinuation of cancer therapy [2, 3]. However, manual tracing on the endocardial border produces inter-observer measurement variabilities that may not be negligible. To overcome this problem, several ultrasound vendors have produced semiautomated LV border detection software that have still limitations due to several reasons.

Recent advances in fully automated software (HeartModel^A.I.^) for left heart chamber quantification with three-dimensional echocardiography (3DE) have been shown promising to quantify LV volumes and LVEF in a routine clinical setting [4–6]. If editing was not performed, the software provides identical numerical values of the measurements on the same 3DE datasets every time. Several studies had determined its accuracy against 3DE manual quantification analysis [4–8] or cardiac magnetic resonance (CMR) as a reference [7, 9–12]. However, it still underestimates LV volumes and overestimate LVEF against CMR [7, 9–12]. The software allows regional and global LV endocardial border editing. Although regional LV endocardial border editing produces a minor change of LV volumes, resulting in the subtle change in LVEF calculation, change of LV endocardial border threshold from 0-point to 100-point makes a remarkable change in LV volumes and LVEF. There are no reports to systematically determine optimal border setting for LV volumes as well as LVEF measurements. Recently, the updated version of the software (Dynamic HeartModel^A.I.^) has been released, and it now provides LV volume curve using 3DE speckle tracking technology.

Accordingly, the aim of this study was to determine optimal LV border threshold at end-diastole (ED) and end-systole (ES) using Dynamic HeartModel^A.I.^ and HeartModel^A.I.^ for the measurements of LVEDV, LVESV, and LVEF against CMR as a reference.

## Methods

### Study population

We retrospectively searched patients who had clinically indicated CMR examination and also agreed to undergo 3DE examination on the same day in University of Occupational and Environmental Health Hospital from January 2017 to July 2018. Among 107 patients for potentially eligible patients, a total of 65 patients who had one-beat 3DE datasets acquisition with specific ultrasound machine that were required for fully automated left chamber quantification software analysis was finally selected for the analysis. All study patients were ethnic Japanese. The ethics committee of the University of Occupational and Environmental Health Hospital approved the study protocol, and informed consent was waived due to the retrospective nature of the analysis.

### 3D echocardiography

Real-time 3DE full-volume datasets were acquired from the apical window with the patient in the left lateral decubitus position using EPIQ 7G scanner (Philips Medical Systems, Andover, MA) equipped with a fully sampled matrix-array transducer (X5-1). The depth and sector angle were adjusted to include the entire left and right chambers. Specific one-beat acquisition mode (3D HMQ) was used to acquire 3DE datasets. Several one-beat acquisitions were performed. These datasets were stored digitally for offline analysis.

### 3D echocardiographic analysis: Automated quantification

#### HeartModel^A.I.^

One-beat 3DE full-volume data sets were analyzed using fully automated quantification software (HeartModel^A.I.^, QLAB, version 10.5, Philips Medical Systems) that detects LV endocardial surfaces using an adaptive analytical algorithm that consists of knowledge-based identification of initial global shape and LV chamber orientation, followed by patient-specific adaptation [7]. After initiating the program, the software automatically determined the ED and ES frames using motion analysis, followed by construction of ED and ES LV casts. The software subsequently determined LVDEV, LVESV, and LVEF without geometric assumptions. To determine the effect of global editing on LV volumes and LVEF, we adjusted ED and ES endocardial border threshold with each increase of threshold value from 0-point to 100-point via 10-point increment, and obtained LVEDV and LVESV at each increment, although adjustments at increments of 1-point are possible. We did not perform any manual editing of endocardial border in every patient and every border setting. LVEF was calculated as LVEDV at specific border threshold—LVESV at specific border threshold divided by LVEDV at specific border threshold multiplied by 100.

This fully automated quantification software produces LV cast based on ultrasound knowledge database, and it occasionally creates erroneous LV cast. Erroneous LV cast constructions are defined if the border of the casts were constructed not on the actual myocardial-blood interface with manual editing correction impossible.

#### Dynamic HeartModel^A.I.^

In contrast to HeartModel that provides LVEDV and LVESV, the new software (Dynamic HeartModel^A.I.^, QLAB version 11.0, Philips Medical Systems) incorporates 3DE speckle tracking technology. It provides LV volume curve during one cardiac cycle. Like HeartModel analysis, we adjusted ED and ES endocardial border threshold with each increase of threshold value from 0-point to 100-point via 10-point increment and obtained LVEDV and LVESV at each increment. Again, we did not perform manual editing. LVEF was calculated as the same process.

### CMR acquisition

CMR imaging was performed with a 3T scanner (Discovery 750W, GE Healthcare) with a phased-array cardiovascular coil. In each patient, retrospective electrocardiographically-gated localizing spin-echo sequences were used to identify the long axis of the heart. Steady state fully precession (SSFP) dynamic gradient-echo cine loops were acquired using retrospective electrocardiographic gating and parallel imaging techniques during 10-sec to 15-sec breath-holds, with a following general parameters: slice thickness of the imaging planes 8 mm, field of view 40×40 cm, scan matrix 200×160, flip angle 50°, repetition/echo times 3.8/1.7 ms, views per segment 20, number of reconstructed cardiac phases 20.

### CMR analysis

CMR LV volumes were measured from multiple short-axis SSFP images using an analytical software (Segment v2.2, Medviso, Lund, Sweden), and the disk-area summation method was used for the calculation of the LVEDV and LVESV. LVEF was measured by standard formula.

### Feature tracking (FT) CMR analysis

CMR images were analyzed using commercial FT software (2D CPA MR; TomTec Imaging Systems, Unterschleissheim, Germany), which is a vector-based analysis tool based on a hierarchical algorithm [13–15]. Using three apical long-axis cine SSFP images, the LV endocardial border at end-systole was semi-automatically drawn after 3-point clicking. Manual adjustment of endocardial border was performed when required. Subsequently, the software automatically propagates the contour and follows its features throughout the cardiac cycle to generate LV volume curve, from which the software provided LVEDV, LVESV and LVEF.

### Statistical analysis

Continuous variables are expressed as mean ± SD or as median and 25^th^ to 75^th^ percentile according to data distribution. All statistical analyses were performed using commercially available software (JMP version 13.1.0, SAS Institute, Cary, NC; R version 3.4.3, The R foundation for Statistical Computing, Vienna, Australia; and Prism 8, GraphPad Software, Inc., La Jolla, CA). Freedman analysis with post-hoc comparison was performed to compare LVEDV, LVESV, and LVEF among different border settings and CMR. Linear correlations and Bland-Altman analysis were performed between the two methods. Since Dynamic HeartModel and CMR FT provide LV volume curves, we determined the reliability of the curves from Dynamic HeartModel against that obtained from CMR FT. Data were extracted using plots digitalization (WebPlotDigitizer), and each curve was digitized and interpolated 100 points during one cardiac cycle. A linear correlation was performed using 100 plots between the two methods, and r-values from linear correlation analysis were calculated in each patient. We calculated coverage probability (CP) as the percentage of the number of measurements whose difference fulfilled pre-defined criteria. Regarding LVEDV and LVESV, the cut-off value was 30 mL between 3DE and CMR measurements [16–18]. Regarding LVEF, the cut-off value was set as 10% between 3DE and CMR determined LVEFs [16–18].

## Results

**Fig 1:**
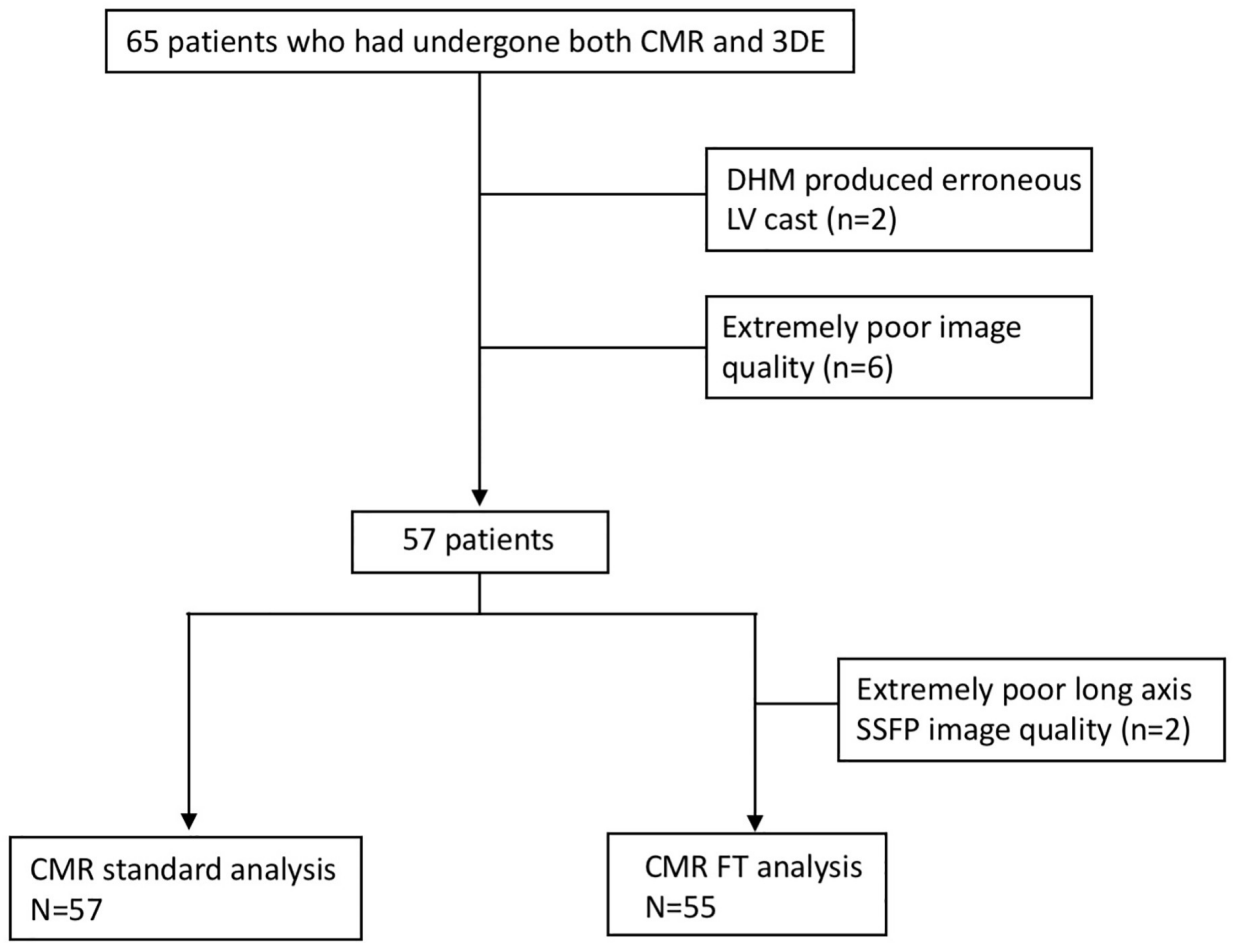
Flow chart of the study population. 3DE, three-dimensional echocardiography; CMR, cardiac magnetic resonance; DHM, Dynamic HeartModel; LV, left ventricular.

Table 1 describes clinical characteristics in study population. 3DE datasets had good image quality in 18%, fair image quality in 45% and poor image quality in 37%, respectively. Among 65 patients, 8 patients were excluded because of no 3DE datasets acquisition due to extremely poor echocardiographic image quality (n = 6) or erroneous LV cast construction (n = 2) by 3DE fully automated software (Feasibility: 88%). Thus, final analysis was conducted in 57 patients. Standard CMR measurements (disk-area summation method) provided LV volumes and LVEF in all patients. CMR FT method provided LV volumes and LVEF in 55 patients (Fig 1). The mean values of LVEF measured on standard CMR method and CMR FT method were 35 ±17% and 35 ±14%.

**Table 1:** Clinical characteristics in study population (n = 65).

Age	71 (64 to 79)
Male/female	39/26
Height (cm)	158 ± 14
Weight (kg)	57 ± 13
BSA (/m^2^)	1.57 ± 0.22
HR at the time of 3DE (bpm)	68 ± 15
HR at the time of CMR (bpm)	66 ± 14
SBP (mmHg)	130 ± 27
DBP (mmHg)	69 ± 12
Clinical diagnosis	
DCMP	5
ICM	11
Secondary cardiomyopathy	22
VHD	9
HCM	2
PH	4
IHD	4
Others	8
Image quality	
Good	12 (18%)
Fair	29 (45%)
Poor	24 (37%)

Continuous data are expressed as mean ± SD or median and interquartile interval.

3DE, three-dimensional echocardiography; BSA, body surface area; CMR, cardiac magnetic resonance; DCMP, dilated cardiomyopathy; DBP, diastolic blood pressure; HCM, hypertrophic cardiomyopathy; HR, heart rate; ICM, ischemic cardiomyopathy; IHD, ischemic heart disease; PH, pulmonary hypertension; SBP, systolic blood pressure; VHD, valvular heart disease

### Comparison of Dyanmic HeartModel^A.I.^ to CMR standard method

**Fig 2:**
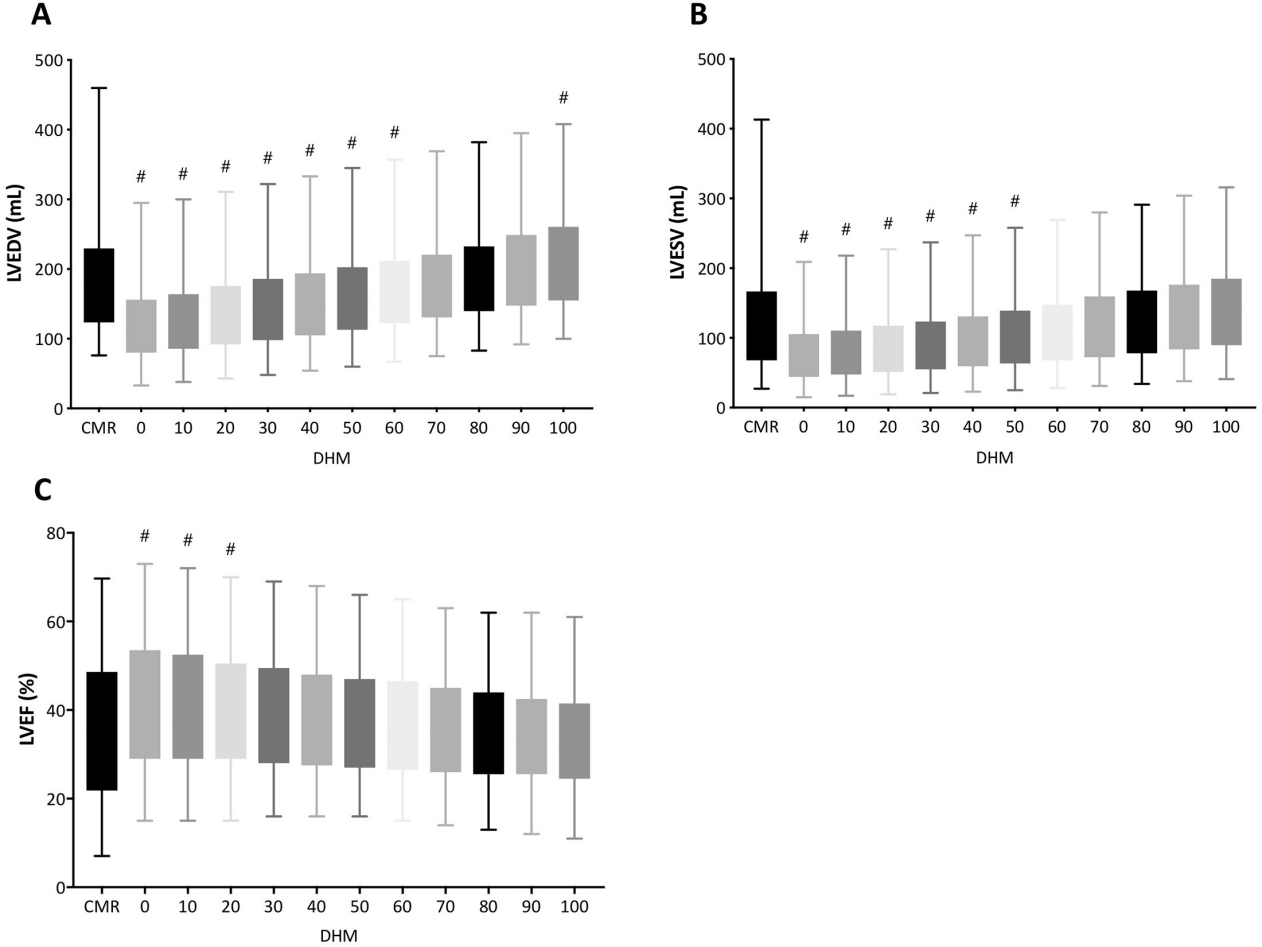
Box and whiskers (minimum to maximum) of left ventricular end-diastolic volume (A), left ventricular end-systolic volume (B), and left ventricular ejection fraction (C) between cardiac magnetic resonance disk-summation method and 3D echocardiography with automated quantification software (Dynamic HeartModel) according to change in border setting. LVEDV, end-diastolic volume; LVEF, left ventricular ejection fraction; LVESV, left ventricular end-systolic volume. #: p<0.05 compared with CMR.

Fig 2 shows LVEDV, LVESV and LVEF among different border threshold using Dynamic HeartModel and corresponding value of CMR disk-area summation method. There were gradual increases in LVEDV and LVESV according to increase of border threshold from 0-point to 100-point. Endocardial border setting from 0-point up to 60-point significantly underestimated LVEDV and border setting of 100-point significantly overestimated LVEDV compared to LVEDV measured by CMR disk-area summation method. Similar tendency was observed in LVESV. LVEF decreased gradually from 0-point to 100-point, if we used the same border threshold of ED and ES.

**Fig 3:**
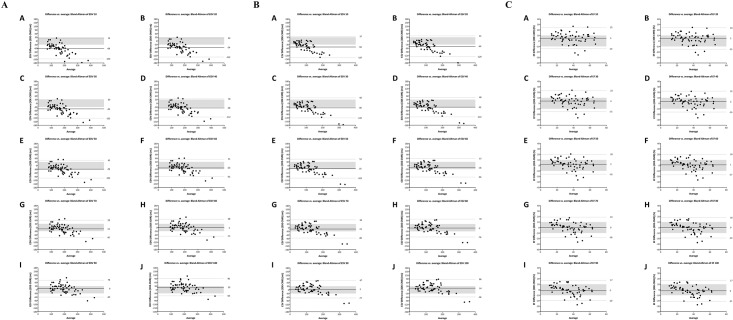
Bland-Altman analysis of left ventricular end-diastolic volume (Fig 3A), left ventricular end-systolic volume (Fig 3B), and left ventricular ejection fraction (Fig 3C) between cardiac magnetic resonance disk-summation method and 3D echocardiography with automated quantification software (Dynamic HeartModel) according to change in border setting from 10 to 100. A, border threshold of 10; B, border threshold of 20; C, border threshold of 30; D, border threshold of 40; E, border threshold of 50; F, border threshold of 60; G, border threshold of 70; H, border threshold of 80: I, border threshold of 90; J, border threshold of 100.

Fig 3A to 3C depict Bland-Altman analysis of LVEDV, LVESV and LVEF between the two methods. Table 2 presents statistical analysis of correlation, bias, 95% limit of agreement (LOA) and CP between Dynamic HeartModel with each border setting and CMR disk-area summation method. Smallest bias of LVEDV, LVESV, and LVEF between CMR and Dynamic HeartModel with high CP value (0.74) of LVEF was observed at border threshold value of 80-point at both ED and ES.

**Table 2:** Comparison of LV volumes and EF between Dynamic HeartModel and CMR disk-area summation method (n = 57).

		CMRstd	DHM 0	DHM 10	DHM 20	DHM 30	DHM 40	DHM 50	DHM 60	DHM 70	DHM 80	DHM 90	DHM 100
LVEDV	median	182	114	121	133	141	147	155	165	175	184	197	208
	25^th^-75^th^	124–230	80–156	86–164	92–176	98–186	105–194	113–203	123–212	131–221	140–233	148–249	155–261
	difference		<0.001	<0.001	<0.001	<0.001	<0.001	<0.001	0.006	1.000	1.000	1.000	0.0082
	r		0.91	0.89	0.91	0.92	0.92	0.92	0.92	0.92	0.92	0.91	0.91
	bias		-68	-62	-54	-46	-38	-29	-20	-11	-1	9	20
	95% LOA		-150 to 14	-147 to 22	-132 to 24	-122 to 30	-112 to 36	-102 to 43	-91 to 51	-81 to 59	-71 to 68	-60 to 78	-50 to 90
	CP		0.16	0.18	0.23	0.32	0.44	0.51	0.60	0.65	0.65	0.65	0.53
LVESV	median	112	72	78	85	92	98	104	109	116	123	132	142
	25^th^-75^th^	68–167	44–106	48–111	51–117	55–124	60–131	64–139	68–148	73–160	78–168	84–177	90–185
	difference		<0.001	<0.001	<0.001	<0.001	<0.001	0.001	0.082	1.000	1.000	1.000	0.1492
	r		0.91	0.92	0.92	0.92	0.92	0.92	0.92	0.92	0.91	0.91	0.91
	bias		-55	-50	-44	-38	-32	-25	-18	-11	-3	5	14
	95% LOA		-144 to 35	-137 to 37	-129 to 41	-120 to 44	-112 to 48	-103 to 52	-94 to 57	-85 to 64	-76 to 70	-67 to 77	-58 to 85
	CP		0.33	0.35	0.46	0.56	0.61	0.58	0.63	0.61	0.67	0.65	0.60
LVEF	median	35	38	37	37	37	37	36	34	35	34	34	32
	25^th^-75^th^	22–49	29–54	29–53	29–51	28–50	28–48	27–47	27–47	26–45	26–44	26–43	25–42
	difference		<0.001	0.003	0.019	0.104	0.349	1.000	1.000	1.000	1.000	1.000	1.000
	r		0.80	0.81	0.81	0.82	0.82	0.83	0.83	0.83	0.83	0.83	0.83
	bias		6.3	5.3	4.5	3.7	2.9	2.1	1.2	0.4	-0.3	-1.1	-1.9
	95% LOA		-13.8 to 26.3	-14.4 to 25.0	-14.6 to 23.6	-15.3 to 22.7	-15.7 to 21.5	-16.3 to 20.5	-17.0 to 19.5	-17.9 to 18.7	-18.7 to 18.2	-19.5 to 17.3	-20.5 to 16.7
	CP		0.58	0.65	0.70	0.72	0.72	0.70	0.72	0.77	0.74	0.74	0.72

25^th^-75^th^, 25^th^ to 75^th^ percentile; CMRstd, cardiac magnetic resonance standard method (disk-area summation method); CP, coverage probability; DHM, dynamic HeartModel; LOA, limit of agreement; LVEDV, left ventricular end-diastolic volume; LVEF, left ventricular ejection fraction; LVESV, left ventricular end-systolic volume.

DHM “x” means DHM using threshold of “x”.

### Comparison of Dyanmic HeartModel^A.I.^ to CMR FT method

**Fig 4:**
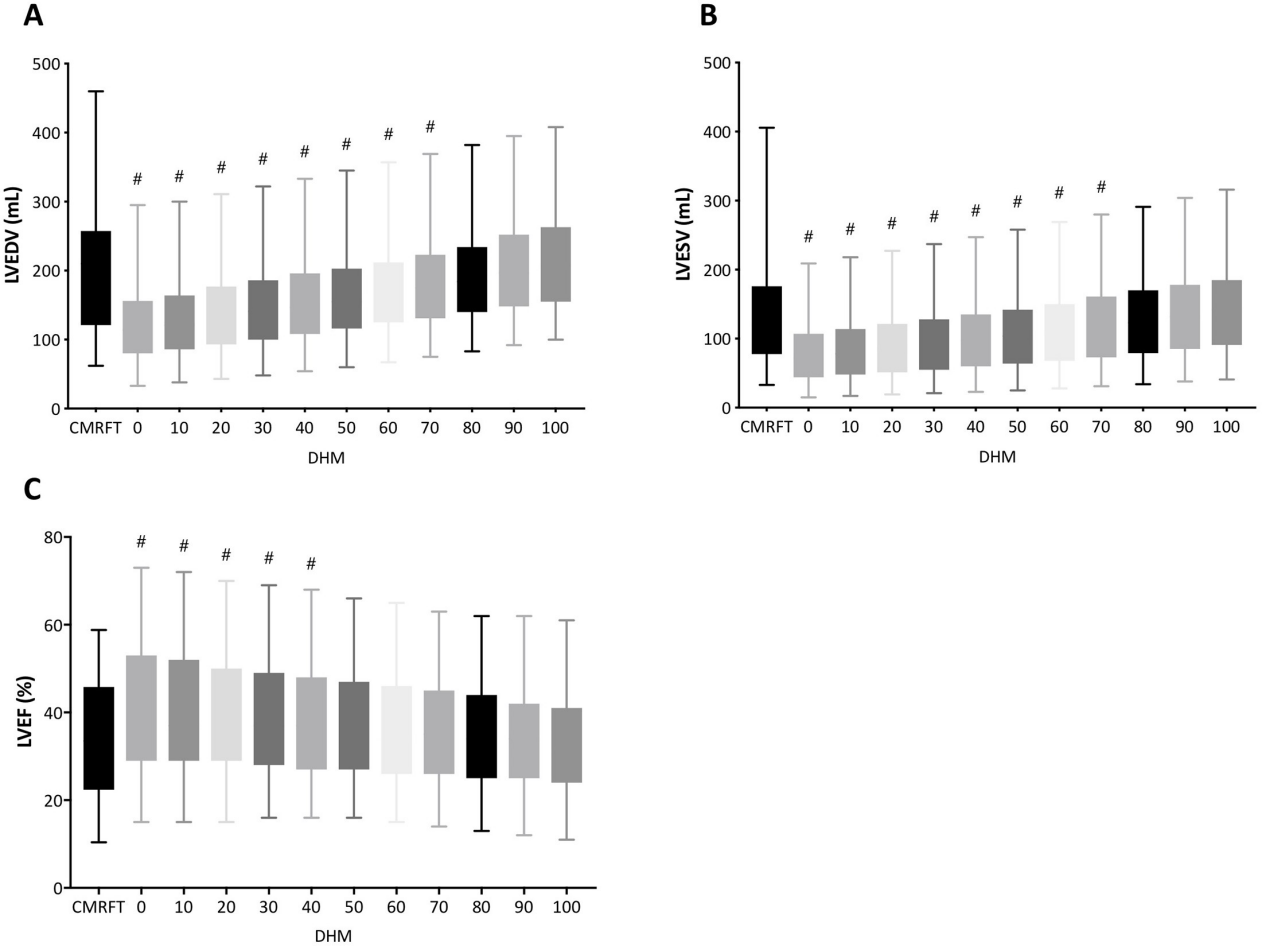
Box and whiskers (minimum to maximum) graphs of left ventricular end-diastolic volume (A), left ventricular end-systolic volume (B), and left ventricular ejection fraction (C) between cardiac magnetic resonance feature tracking and 3D echocardiography with automated quantification software (Dynamic HeartModel) according to change in border setting.

Fig 4 shows LVEDV, LVESV and LVEF among different border threshold using Dynamic HeartModel and corresponding value of CMR FT method. Endocardial border setting from 0-point up to 70-point significantly underestimated both LVEDV and LVESV compared with corresponding values measured by CMR FT method. There were no significant differences in LVEDV and LVESV between Dynamic HeartModel with border setting from 80-point to 100-point and CMR FT method.

**Fig 5:**
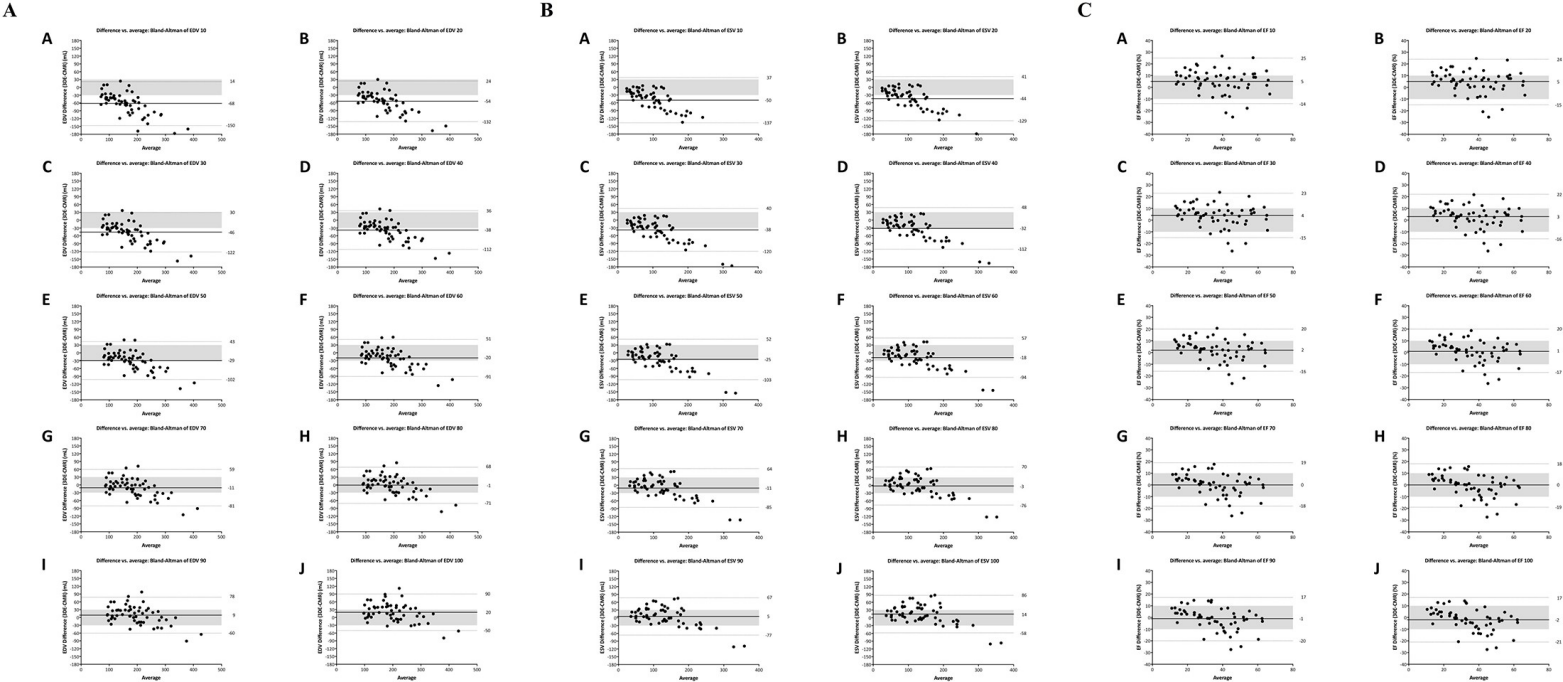
Bland-Altman analysis of left ventricular end-diastolic volume (Fig 5A), left ventricular end-systolic volume (Fig 5B), and left ventricular ejection fraction (Fig 5C) between cardiac magnetic resonance feature tracking method and 3D echocardiography with automated quantification software (Dynamic HeartModel) according to change in border setting from 10 to 100. A, border threshold of 10; B, border threshold of 20; C, border threshold of 30; D, border threshold of 40; E, border threshold of 50; F, border threshold of 60; G, border threshold of 70; H, border threshold of 80: I, border threshold of 90; J, border threshold of 100.

Fig 5A to 5C depict Bland-Altman analysis of LVEDV, LVESV and LVEF between the two methods. Table 3 represents statistical analysis of correlation, bias, 95% LOA and CP between Dynamic HeartModel with each border setting and CMR FT method. Smallest bias of LVEDV, LVESV, and LVEF between CMR and Dynamic HeartModel with high CP value (0.84) of LVEF was observed at border threshold value of 90-point at both ED and ES.

**Table 3:** Comparison of LV volumes and EF between Dynamic HeartModel and CMR FT method (n = 55).

		CMR FT	DHM 0	DHM 10	DHM 20	DHM 30	DHM 40	DHM 50	DHM 60	DHM 70	DHM 80	DHM 90	DHM 100
LVEDV	median	210	114	121	133	141	147	155	165	175	184	197	208
	25^th^-75^th^	121–257	80–156	86–164	93–177	100–186	108–196	116–203	125–212	131–223	140–234	148–252	155–263
	difference		<0.001	<0.001	<0.001	<0.001	<0.001	<0.001	<0.001	0.005	0.950	1.000	1.000
	r		0.93	0.91	0.94	0.94	0.94	0.94	0.94	0.94	0.94	0.93	0.93
	bias		-79	-74	-65	-57	-49	-40	-31	-22	-12	-2	9
	95% LOA		-161 to 2	-158 to 10	-142 to 11	-131 to 17	-121 to 23	-110 to 30	-99 to 37	-89 to 45	-78 to 54	-67 to 64	-57 to 76
	CP		0.13	0.15	0.2	0.25	0.35	0.4	0.4	0.47	0.6	0.65	0.62
LVESV	median	119	72	78	85	92	98	104	109	116	123	132	142
	25^th^-75^th^	78–176	44–107	48–114	51–121	55–128	60–135	64–142	68–150	73–161	79–170	85–178	91–185
	difference		<0.001	<0.001	<0.00	<0.001	<0.001	<0.001	<0.001	0.021	1.000	1.000	1.000
	r		0.94	0.94	0.94	0.94	0.95	0.95	0.95	0.94	0.94	0.94	0.93
	bias		-63	-57	-52	-46	-39	-33	-26	-18	-10	-2	7
	95% LOA		-145 to 20	-137 to 22	-129 to 25	-120 to 28	-111 to 32	-101 to 36	-92 to 41	-83 to 47	-73 to 53	-64 to 60	-55 to 69
	CP		0.25	0.31	0.33	0.42	0.49	0.56	0.64	0.67	0.69	0.73	0.64
LVEF	median	33.5	38	37	37	37	37	36	34	35	34	34	32
	25^th^-75^th^	22–46	29–53	29–52	29–50	28–49	27–48	27–47	26–46	26–45	25–44	25–42	24–41
	difference		<0.001	<0.001	<0.001	0.003	0.036	0.326	1.000	1.000	1.000	1.000	1.000
	r		0.86	0.87	0.87	0.87	0.87	0.88	0.88	0.87	0.87	0.87	0.86
	bias		6.6	5.7	4.8	4.0	3.2	2.5	1.6	0.8	0.1	-0.7	-1.5
	95% LOA		-8.9 to 22.1	-9.2 to 20.6	-9.5 to 19.2	-10.0 to 18.1	-10.5 to 17.0	-11.0 to 15.9	-11.7 to 15.0	-12.7 to 14.2	-13.5 to 13.7	-14.4 to 13.0	-15.7 to 12.7
	CP		0.71	0.76	0.78	0.82	0.82	0.82	0.87	0.89	0.84	0.84	0.84

Data are expressed as median and 25^th^ to 75^th^ percentile. CMR, cardiac magnetic resonance; CP, coverage probability; DHM, dynamic HeartModel; FT, feature tracking; LOA, limit of agreement; LVEDV, left ventricular end-diastolic volume; LVEF, left ventricular ejection fraction; LVESV, left ventricular end-systolic volume.

DHM “x” means DHM using threshold of “x”.

**Fig 6:**
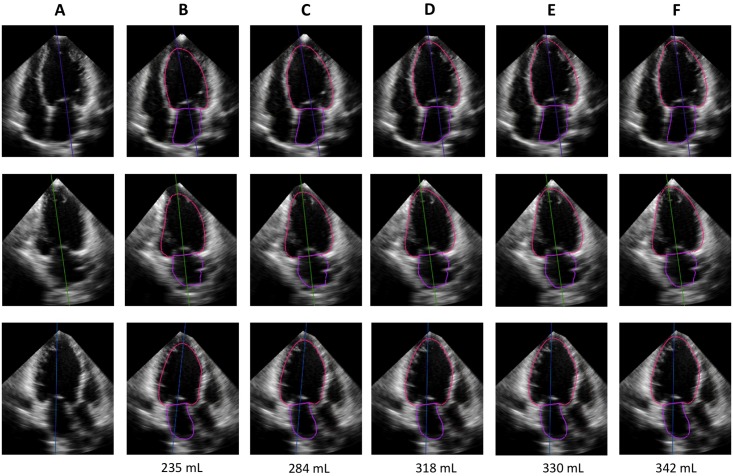
Left ventricular end-diastolic endocardial border tracing line used by different LV endocardial border threshold in a representative case. Upper, middle, and lower panels show apical 4-chamber, 2-chamber, and long-axis views extracted from 3D dataset. A, no border line; B, border threshold of 0; C, border threshold of 50; D, border threshold of 80; E, border threshold of 90; F, border threshold of 100.

Fig 6 shows ED LV border tracing line used by different LV endocardial border threshold in a representative case.

**Fig 7:**
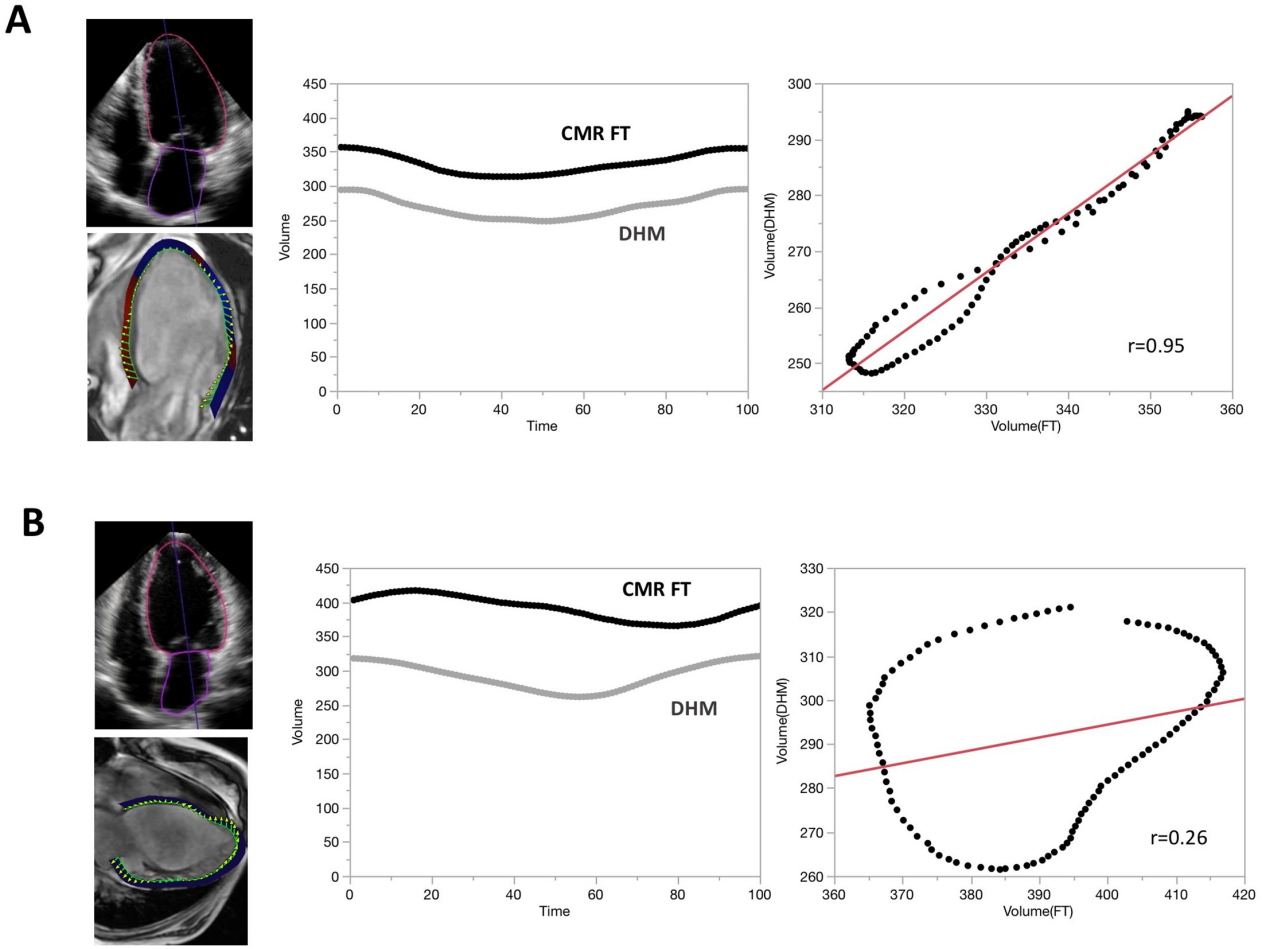
Representative cases for left ventricular (LV) volume curves generated by fully automated quantification software and cardiac magnetic resonance (CMR) feature tracking method. A: A case of good correlation. Left upper panel shows apical 4-chamber view of Dynamic HeartModel and left lower panel shows apical 4-chamber view of CMR feature tracking. Middle panel shows LV volume curves by both methods. Right panel shows their correlation. From LV volume curves during one cardiac cycle, we extracted 100 data points using interpolation and digitalization of the curve. B: A case of poor correlation. Note timing of maximum and minimum LV volume is different.

Fig 7 represents 2 cases with LV volume curves from Dynamic HeartModel and CMR FT and their correlations. Upper case shows good correlation (r = 0.95) and lower case shows poor correlation (r = 0.26) of LV volume curves between the two modalities. Overall comparison showed that a median value of r was 0.87 (25^th^ to 75^th^ percentile: 0.80 to 0.93) in 55 patients.

### Comparison of HeartModel^A.I.^ to CMR

S1 and S2 Tables show analysis of correlation, bias, 95% LOA and CP between HeartModel with serial border setting and CMR disk-area summation method (S1 Table) and between HeartModel and CMR FT method (S2 Table). Smallest bias of LVEDV, LVESV, and LVEF between CMR disk-area summation method and HeartModel with high CP value (0.67) of LVEF was observed at border threshold value of 80 at both ED and ES. Smallest bias of LVEDV, LVESV, and LVEF between CMR FT method and HeartModel with high CP value (0.82) of LVEF was observed at border threshold value of 90 at both ED and ES. However, observed CP values of LVEDV, LVESV and LVEF were lower than that observed using Dynamic HeartModel.

### Comparison of Dyanmic HeartModel^A.I.^ to HeartModel^A.I.^

**Fig 8:**
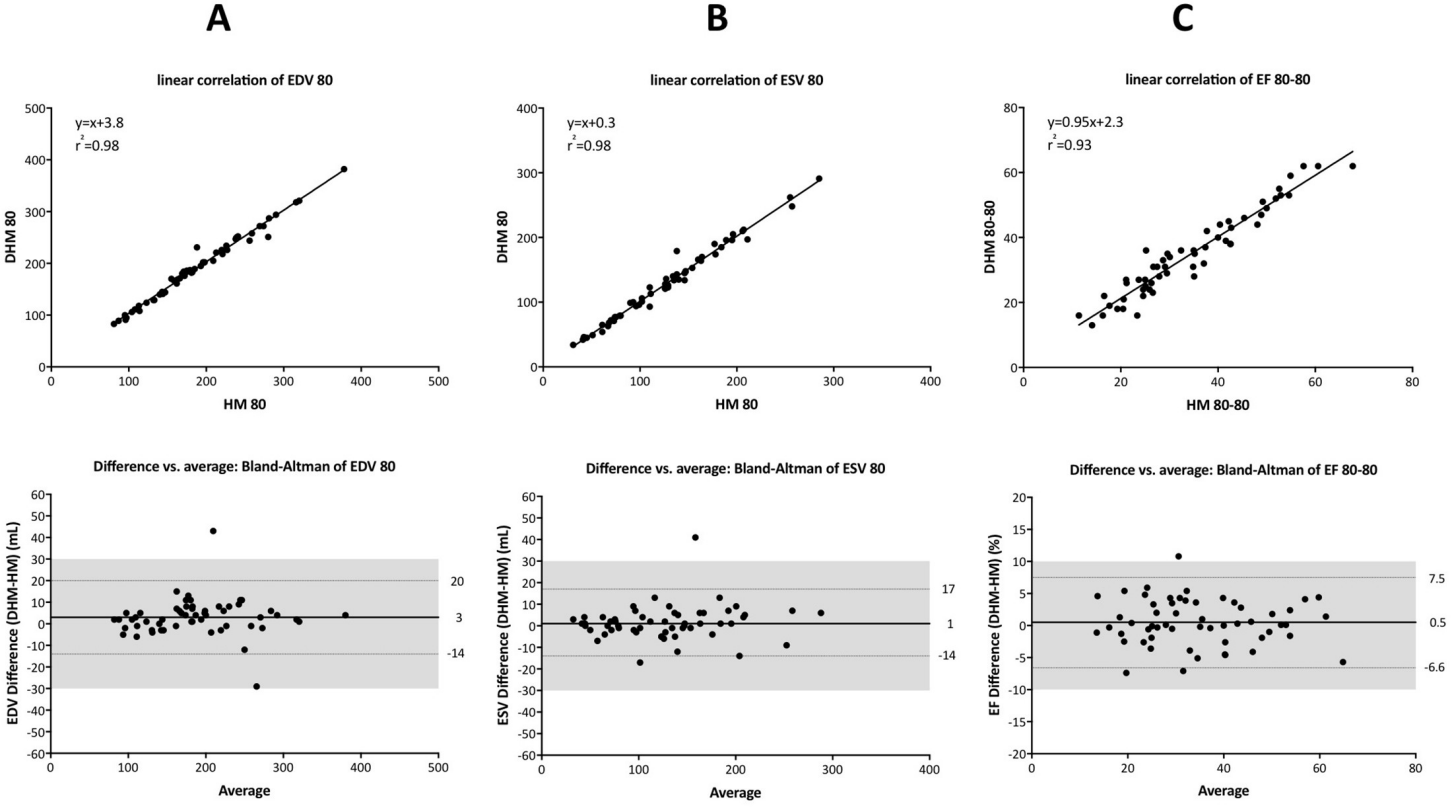
A linear correlation (upper panels) and Bland-Altman analysis (lower panels) of left ventricular end-diastolic volume (A), left ventricular end-systolic volume (B), and left ventricular ejection fraction (C) between Dynamic HeartModel and HeartModel with use of a border threshold value of 80 at both end-diastole and end-systole.

Fig 8 showed linear correlation and Bland-Altman analysis of LVEDV, LVESV, and LVEF between Dynamic HeartModel and HeartModel with use of a border threshold value of 80 at both ED and ES. Although two methods showed tight linear correlations, measurement values were different in majority of cases.

## Discussion

This is the first systematic study to investigate optimal endocardial border threshold values for determination of LV volumes and LVEF using 3DE fully automated left chamber quantification software (Dynamic HeartModel) and its earlier version (HeartModel) against CMR as a reference. Our results showed that the bias between both Dynamic HeartModel and HeartModel determined LVEDV and LVEDV were smallest when the border threshold value was set to 80-point against CMR disk-area summation method, or 90-point when compared to CMR FT method. Dynamic HeartModel could improve measurement accuracy over previous version of the software because the former was yielded with higher values of CP with equivalent border setting.

### Previous studies

The importance of cardiac chamber size and function in cardiac imaging cannot be over-emphasized. The limitations of 2DE have been inexorably mentioned in previous studies, including foreshortening and geometric assumptions [19–21]. The Recommendations for Cardiac Chamber Quantification by Echocardiography in Adults from American Society of Echocardiography and European Association of Cardiovascular Imaging suggested although LV size should be routinely assessed by 2DE, 3DE measurement and reporting of LV volumes is recommended when feasible [22]. Despite the good correlation with CMR, several previous studies using manual or semi-automated contour method showed underestimation of 3DE derived-LV volumes against CMR [19, 20]. With the 3DE gaining popularity and momentum, efforts have been made to integrate its practice into daily routine of ultrasound laboratories. However, previous software were at best semi-automatic and often manual adjustment were required, true border delineation often necessitates additional extra times with observer variabilities that cannot be negligible. Therefore, even though 3DE offers significant improvements, there are impediments to its adoption into clinical practice due to tedious manual editing.

Fully automated left chamber quantification software with knowledge-based technology utilizes 3DE datasets with less technical demanding steps, and the time required to analyze 3DE LV volumes and LVEF is only less than 30 seconds without any manual editing [4, 7]. Time required for the analysis is quite short compared with that using standard CMR disk-summation method (5–10 minutes) or CMR FT method (2–3 minutes). The software allows global and regional editing to correct endocardial border. The global editing permits adjustment of the border threshold value from 0-point to 100-point on ED and ES borders to obtain an LV casts that fit the visual assessment. Previous validation studies consistently reported that 3DE with fully automated quantification software underestimate LVEDV (-13 mL to -30 mL) and LVESV (-7 mL to -29 mL) against CMR as a reference, albeit each author used different LV endocardial border settings [7, 9–12]. It is well known that the accurate endocardial border setting is essential for the accurate measurement of LV volumes and LVEF. However, there are still no recommendations regarding the optimal endocardial border threshold values that can be routinely employed for clinical practice.

### Current study

In this study, feasibility of LV volumes and LVEF measurements was 88%, and the values were in agreement with the previous studies [4, 6]. Fully automated quantification software is designed and trained to recognize two myocardial borders—the inner and outer extents of the myocardial tissue—those being at the blood-tissue interface and at the interface of the non-compacted and compacted myocardium. Border line can be changed according to global border threshold setting. We investigated how the ED and ES border threshold value setting in fully automated left chamber quantification software affected our quantification of LV volumes and LVEF, in comparison to the measurement by CMR as references. In addition to standard disk-area summation method with serial SSFP short axis images, we also applied CMR FT analysis in this study. CMR FT analysis is another method of choice to quantify LV function. Since FT analysis uses 3 apical views to generate LV volumes and LVEF like echocardiography, the comparison between the two methods are more appropriate. It also provides LV volume curves from 3 apical SSFP images. Thus, it had an opportunity to compare LV volume curves derived from Dynamic HeartModel and CMR FT. As seen in Figs 2 and 4, we found there was parallel increase in LVEDV and LVESV and corresponding parallel decrease in LVEF when ED and ES endocardial border threshold were adjusted simultaneously from value of 0-point to 100-point at increment of 10-point. We observed that larger border threshold values allowed LV volumes to closely approximate volumes obtained by CMR. These findings re-affirm the fact that previous routine echocardiographic endocardial border delineations usually result in smaller sizes of LV volumes compared to CMR quantifications, if echocardiographers do not specifically adjust outwardly their manual LV border tracing. Our results showed that the bias of LVEDV and LVESV between both Dynamic HeartModel/HeartModel and CMR disk-area summation method were smallest when the border threshold value was set to 80, or 90 when compared to CMR FT method. Using border threshold value 80 or 90 at both ED and ES for determination of LVEF, the bias between fully automated quantification software and CMR was also smallest with acceptable CP values. Higher CP value of Dynamic HeartModel over HeartModel supports improved measurement accuracy, and this could be relating to updated algorithm of the software.

The Dynamic HeartModel performs 3DE speckle tracking on the LV endocardial border in each frame over the entire cardiac cycle, resulting in the generation of LV volume curves. We determined its reliability against corresponding LV volume curves derived from CMR FT method. Median r -values by linear regression analysis from the whole subjects (r = 0.87) supported its accuracy and reliability.

In summary, this is the first study to perform systematically the determination of optimal border threshold by fully automated left chamber quantification software for the quantification of LV volumes and LVEF compared to the standard CMR disk-area summation method and the novel CMR FT method. The fully automated quantification software with appropriate border threshold value setting has a potential for the evaluation of LV function with confidence.

### Study limitations

There were several limitations that should be acknowledged in this study. First, the software was not associated with 100% feasibility. In this study, there were 2 cases of incorrect LV casts that could not be edited, and other 6 patients were excluded due to very poor image quality rejected 3DE datasets acquisition, resulting in overall feasibility of 88%. The number however, was similar to previously performed feasibility studies [6]. Second, we only investigated ED and ES border threshold settings at every 10-point basis. Third, to determine the reliability of LV volume curves by Dynamic HeartModel, we used corresponding LV volume curve using CMR FT that has not been validated as a reference standard. Fourth, temporal resolution of CMR (76ms) used in this study was above the Society for Cardiovascular Magnetic Resonance recommendations for LV function assessment [23]. It may overestimate LVESV, resulting in some underestimation of LVEF. Last, further study should be required to validate our results in the larger number of study patients with multicenter setting.

## Conclusion

We concluded that LV endocardial border threshold value can be set around 80 to 90 with the same number of LV ED and ES threshold to approximate LVEDV, LVESV and LVEF with clinically acceptable CP values of LVEF as CMR values as a reference.

## Supporting information

S1 Table Analysis of correlation, bias, 95% LOA and CP between HeartModel with serial border setting and CMR disk-area summation method. (DOCX)

S2 Table Analysis of correlation, bias, 95% LOA and CP between HeartModel with serial border setting and CMR FT method. (DOCX)
