# Correction: Optimal threshold of three-dimensional echocardiographic fully automated software for quantification of left ventricular volumes and ejection fraction: Comparison with cardiac magnetic resonance disk-area summation method and feature tracking method

**DOI:** 10.1371/journal.pone.0214781

**Published:** 2019-03-28

**Authors:** The PLOS ONE Staff

The first and fifth authors’ initials appear incorrectly in the citation. The correct citation is: Wu VC, Kitano T, Nabeshima Y, Otani K, Chu PH, Takeuchi M (2019) Optimal threshold of three-dimensional echocardiographic fully automated software for quantification of left ventricular volumes and ejection fraction: Comparison with cardiac magnetic resonance disk-area summation method and feature tracking method. PLoS ONE 14(1): e0211154. https://doi.org/10.1371/journal.pone.0211154. The publisher apologizes for the error.
