# Supplementary material for: Optimal threshold of three-dimensional echocardiographic fully automated software for quantification of left ventricular volumes and ejection fraction: Comparison with cardiac magnetic resonance disk-area summation method and feature tracking method
Source: PLoS One. 2019 Jan 28;14(1):e0211154. doi: 10.1371/journal.pone.0211154 (PMC6349335; doi:10.1371/journal.pone.0211154)
Supplement: S1 Table — (DOCX) [file pone.0211154.s001.docx]

S1 Table. Comparison of LV volumes and EF between HeartModel and CMR disk-area summation method (n=57)

|  |  | CMRstd | HM 0 | HM 10 | HM 20 | HM 30 | HM 40 | HM 50 | HM 60 | HM 70 | HM 80 | HM 90 | HM 100 |
| --- | --- | --- | --- | --- | --- | --- | --- | --- | --- | --- | --- | --- | --- |
| LVEDV | median | 182 | 112 | 116 | 125 | 132 | 141 | 149 | 157 | 166 | 179 | 188 | 199 |
|  | 25^th^-75^th^ | 126-230 | 75-156 | 81-161 | 88-173 | 96-180 | 104-188 | 112-196 | 120-204 | 128-215 | 137-227 | 149-239 | 159-252 |
|  | difference |  | <0.001 | <0.001 | <0.001 | <0.001 | <0.001 | <0.001 | <0.001 | 0.100 | 1.000 | 1.000 | 0.032 |
|  | r |  | 0.90 | 0.92 | 0.91 | 0.91 | 0.92 | 0.92 | 0.92 | 0.92 | 0.92 | 0.92 | 0.92 |
|  | bias |  | -72 | -67 | -58 | -50 | -42 | -33 | -26 | -15 | -5 | 5 | 16 |
|  | 95% LOA |  | -158 to 13 | -159 to 24 | -138 to 22 | -127 to 27 | -117 to 32 | -105 to 39 | -104 to 52 | -83 to 53 | -71 to 61 | -60 to 70 | -49 to 81 |
|  | CP |  | 0.12 | 0.14 | 0.21 | 0.26 | 0.35 | 0.42 | 0.51 | 0.60 | 0.70 | 0.70 | 0.60 |
| LVESV | median | 112 | 66 | 72 | 78 | 85 | 92 | 100 | 109 | 117 | 126 | 135 | 143 |
|  | 25^th^-75^th^ | 68-167 | 37-102 | 41-108 | 46-113 | 51-119 | 56-126 | 60-133 | 65-142 | 70-151 | 75-162 | 80-171 | 88-180 |
|  | difference |  | <0.001 | <0.001 | <0.001 | <0.001 | <0.001 | <0.001 | 0.019 | 1.000 | 1.000 | 1.000 | 0.269 |
|  | r |  | 0.92 | 0.92 | 0.92 | 0.92 | 0.92 | 0.92 | 0.92 | 0.92 | 0.91 | 0.91 | 0.90 |
|  | bias |  | -58 | -53 | -47 | -41 | -35 | -28 | -21 | -13 | -5 | 4 | 13 |
|  | 95% LOA |  | -147 to 32 | -140 to 34 | -132 to 38 | -123 to 41 | -115 to 45 | -106 to 50 | -97 to 55 | -88 to 62 | -79 to 69 | -69 to 77 | -60 to 86 |
|  | CP |  | 0.30 | 0.35 | 0.39 | 0.49 | 0.60 | 0.61 | 0.61 | 0.58 | 0.63 | 0.65 | 0.65 |
| LVEF | median | 35 | 40 | 39 | 37 | 36 | 36 | 36 | 35 | 35 | 32 | 30 | 29 |
|  | 25^th^-75^th^ | 22-51 | 29-56 | 29-54 | 28-53 | 27-51 | 26-49 | 26-48 | 25-46 | 25-45 | 25-43 | 25-43 | 24-42 |
|  | difference |  | <0.001 | 0.007 | 0.009 | 0.080 | 0.832 | 1.000 | 1.000 | 1.000 | 1.000 | 1.000 | 1.000 |
|  | r |  | 0.82 | 0.83 | 0.83 | 0.83 | 0.83 | 0.83 | 0.82 | 0.82 | 0.81 | 0.80 | 0.79 |
|  | bias |  | 6.6 | 4.4 | 4.9 | 3.9 | 2.9 | 2.0 | 0.2 | 0.1 | -0.7 | -1.7 | -2.6 |
|  | 95% LOA |  | -12.4 to 25.6 | -24.5 to 33.2 | -13.7 to 23.4 | -14.5 to 22.3 | -15.5 to 21.4 | -16.3 to 20.4 | -22.7 to 23.1 | -18.9 to 19.1 | -20.1 to 18.6 | -21.5 to 18.1 | -22.9 to 17.7 |
|  | CP |  | 0.60 | 0.60 | 0.61 | 0.67 | 0.67 | 0.67 | 0.63 | 0.67 | 0.67 | 0.68 | 0.65 |

25^th^-75^th^, 25^th^ to 75^th^ percentile; CMRstd, cardiac magnetic resonance standard method (disk-area summation method); CP, coverage probability; HM, HeartModel; LOA, limit of agreement; LVEDV, left ventricular end-diastolic volume; LVEF, left ventricular ejection fraction; LVESV, left ventricular end-systolic volume.

HM “x” means HM using threshold of “x”.
