# Supplementary material for: Optimal threshold of three-dimensional echocardiographic fully automated software for quantification of left ventricular volumes and ejection fraction: Comparison with cardiac magnetic resonance disk-area summation method and feature tracking method
Source: PLoS One. 2019 Jan 28;14(1):e0211154. doi: 10.1371/journal.pone.0211154 (PMC6349335; doi:10.1371/journal.pone.0211154)
Supplement: S2 Table — (DOCX) [file pone.0211154.s002.docx]

S2 Table. Comparison of LV volumes and EF between HeartModel and CMR FT method (n=55)

|  |  | CMR FT | HM 0 | HM 10 | HM 20 | HM 30 | HM 40 | HM 50 | HM 60 | HM 70 | HM 80 | HM 90 | HM 100 |
| --- | --- | --- | --- | --- | --- | --- | --- | --- | --- | --- | --- | --- | --- |
| LVEDV | median | 210 | 112 | 116 | 125 | 132 | 141 | 149 | 157 | 166 | 179 | 188 | 199 |
|  | 25^th^-75^th^ | 121-257 | 77-156 | 84-163 | 91-175 | 99-182 | 107-188 | 114-197 | 120-205 | 130-215 | 140-227 | 151-241 | 159-255 |
|  | difference |  | <0.001 | <0.001 | <0.001 | <0.001 | <0.001 | <0.001 | <0.001 | <0.001 | 0.112 | 1.000 | 1.000 |
|  | r |  | 0.92 | 0.92 | 0.93 | 0.93 | 0.93 | 0.94 | 0.94 | 0.94 | 0.93 | 0.93 | 0.93 |
|  | bias |  | -84 | -79 | -69 | -61 | -53 | -44 | -37 | -26 | -16 | -5 | 6 |
|  | 95% LOA |  | -169 to 1 | -170 to 13 | -148 to 10 | -138 to 15 | -127 to 21 | -116 to 27 | -116 to 42 | -93 to 41 | -82 to 51 | -70 to 60 | -60 to 72 |
|  | CP |  | 0.07 | 0.11 | 0.18 | 0.23 | 0.28 | 0.35 | 0.39 | 0.39 | 0.54 | 0.63 | 0.56 |
| LVESV | median | 119 | 66 | 72 | 78 | 85 | 92 | 100 | 109 | 117 | 126 | 135 | 143 |
|  | 25^th^-75^th^ | 78-176 | 38-101 | 41-107 | 46-113 | 52-120 | 57-128 | 61-135 | 66-143 | 70-151 | 75-163 | 80-172 | 88-180 |
|  | difference |  | <0.001 | <0.001 | <0.001 | <0.001 | <0.001 | <0.001 | <0.001 | 0.003 | 1.000 | 1.000 | 1.000 |
|  | r |  | 0.95 | 0.95 | 0.95 | 0.95 | 0.95 | 0.95 | 0.94 | 0.94 | 0.94 | 0.93 | 0.93 |
|  | bias |  | -66 | -61 | -55 | -49 | -42 | -35 | -28 | -20 | -12 | -3 | 6 |
|  | 95% LOA |  | -148 to 16 | -140 to 19 | -131 to 22 | -123 to 25 | -114 to 29 | -105 to 34 | -95 to 39 | -86 to 45 | -77 to 52 | -67 to 60 | -59 to 70 |
|  | CP |  | 0.21 | 0.25 | 0.30 | 0.37 | 0.44 | 0.51 | 0.60 | 0.67 | 0.67 | 0.68 | 0.65 |
| LVEF | median | 33.5 | 40 | 44 | 37 | 36 | 36 | 36 | 35 | 35 | 32 | 30 | 29 |
|  | 25^th^-75^th^ | 22-44 | 29-54 | 33-57 | 28-51 | 27-50 | 26-48 | 25-48 | 25-46 | 25-44 | 25-43 | 24-42 | 24-42 |
|  | difference |  | <0.001 | <0.001 | <0.001 | 0.004 | 0.041 | 0.400 | 1.000 | 1.000 | 1.000 | 1.000 | 1.000 |
|  | r |  | 0.86 | 0.87 | 0.87 | 0.87 | 0.87 | 0.87 | 0.87 | 0.86 | 0.85 | 0.84 | 0.83 |
|  | bias |  | 7.1 | 9.2 | 5.4 | 4.5 | 3.5 | 2.6 | 0.8 | 0.7 | -0.1 | -1.1 | -2.0 |
|  | 95% LOA |  | -8.2 to 22.4 | -17.4 to 35.6 | -9.1 to 19.8 | -9.6 to 18.6 | -10.5 to 17.4 | -11.2 to 16.4 | -18.9 to 20.5 | -13.4 to 14.8 | -14.5 to 14.2 | -15.9 to 13.7 | -17.3 to 13.3 |
|  | CP |  | 0.61 | 0.63 | 0.67 | 0.72 | 0.74 | 0.77 | 0.77 | 0.81 | 0.84 | 0.82 | 0.79 |

Data are expressed as median and 25^th^ to 75^th^ percentile. CMR, cardiac magnetic resonance; CP, coverage probability; FT, feature tracking; HM, HeartModel; LOA, limit of agreement; LVEDV, left ventricular end-diastolic volume; LVEF, left ventricular ejection fraction; LVESV, left ventricular end-systolic volume.

HM “x” means HM using threshold of “x”.
